# Assessment of renal transplant dysfunction by doppler sonography: A systematic review and meta-analysis

**DOI:** 10.22088/cjim.15.3.374

**Published:** 2024-08-01

**Authors:** Shirin Habibi, Seyed Morteza Bagheri, Mostafa Ghadamzadeh, Seyed Reza Saadat Mostafavi, Adeleh Dadkhah

**Affiliations:** 1 Department of Radiology, School of Medicine, Iran University of Medical Sciences, Tehran, Iran; 2 Department of Radiology, Hasheminejad Kidney Center (HKC), School of Medicine, Iran University of Medical Sciences, Tehran, Iran

**Keywords:** Ultrasonography, Renal artery stenosis, Resistive index, Meta-analysis

## Abstract

**Background:**

Doppler sonography parameters, particularly the resistive index (RI), have been identified as an essential tool for assessing renal transplant dysfunction (RTD). However, there is some ambiguity in the findings of previous research studies on this matter. Therefore, the objective of our study is to examine the relationship between changes in RI subsequent to RTD.

**Methods:**

This was a systematic review and meta-analysis study. We searched three electronic databases PubMed, Web of Science, and Scopus, from the year 2000 to 10 May 2022. The main effect size was considered as the mean RI differences of cases with RTD confirmed by biopsy with control patients with no RTD. We used random effect models to pool the effect size.

**Results:**

Thirteen studies were included in our review. The pooled mean (95% CI) for the control group was calculated to be 0.71 (0.67, 0.75) and for patients with renal transplant dysfunction was 0.73 (0.68, 0.78), under a random effect model with high heterogeneity for both analyses (I^2^=98% and 97%, respectively). The pooled mean was significantly different between the control group and patients with RTD (P= 0.05), based on a t-test of pooled effect sizes.

**Conclusions:**

Based on the result of our study, we showed that there is a significant difference between RI in patients with kidney transplant dysfunction and the control group. However, RI cannot substitute kidney biopsy in the management and diagnosis of RTD.

The most frequent reasons for renal transplant dysfunction (RTD) are acute rejection, chronic rejection, and acute tubular necrosis (1). Clinically, it may be difficult to distinguish between these entities. As the number of accomplished renal transplants and the survival rate of these patients rises, providing a noninvasive, accurate approach to identifying the etiology of RTD has become crucial (2, 3). Obtaining histopathologic information about a dysfunctional renal transplant is crucial, and one effective method for doing so is through a kidney biopsy.

 However, it is important to note that this technique is invasive and may not be suitable for individuals with certain conditions, such as coagulopathy. Additionally, there is a risk of complications from the biopsy, such as the formation of large perinephric hematomas, which can cause or worsen renal failure (4). Sonography has become a standard procedure for assessing renal transplants, as it is a well-established noninvasive imaging examination (5). 

On gray-scale imaging of a transplant kidney with malfunction, some morphologic alterations, such as size, parenchymal echogenicity, and corticomedullary differentiation, may be noticed.

However, one important issue is that the assessment of renal dysfunction with sonography lack specificity (6). Furthermore, it has been noted that renal sonographic changes appear considerably later than pathological changes, such as an elevated blood creatinine level, once there is already a problem with intrarenal circulation (6). The resistive index (RI) has been used to assess the hemodynamics of both transplanted and natural kidneys (7, 8). 

The RI has been reported to be a useful Doppler sonography index in the evaluation of kidney transplant dysfunction in certain investigations. (9, 10), while other research studies have proven equivocally (11, 12). Finally, some publications have shown that comparing follow-up and initial RI values might be beneficial for tracking allograft development, evaluating treatment success, and diagnosing subclinical atherosclerotic damage in transplant recipients' cardiovascular systems. (13). Thus, this study aims at assessing the association of alterations in RI with histopathologic changes in RTDs. 

## Methods

This study is based on the Preferred Reporting Items for Systematic Reviews and Meta-Analyses (PRISMA) (14). The targeted outcomes were the mean of RI in patients with RTD and the mean of RI in the control group. We searched two international databases (PubMed, Scopus, and Web of Science) for this purpose from the year 200 up to 10 May 2022. 

The following keywords were used for searching databases: 1) Kidney transplant, renal transplant, kidney allograft, or renal allograft; 2) Doppler sonography or Doppler ultrasound (Appendix I). No language or type of publication limitation was applied.

**Eligibility assessment:** We included studies that reported data on the mean of RI in both the control group and patients with RTD. Cross-sectional analytical, cohort or case-control studies were included in our study. The RTD had to be diagnosed based on the biopsy or arteriographic method, and biopsy specimens that were not diagnostic for RTD were regarded as controls in the included studies. Studies that contain one or more of the following criteria were excluded: 1) published before 2000, 2) case report or series studies, systematic reviews or meta-analysis studies, grey literature, 3) Studies without a control group, 4) non-English full-texts, and 5) not reported RI as the index of interest. 

**Literature review and data extraction:** The titles and abstracts of all records which were found in PubMed or Scopus were screened independently by two reviewers. Selected full texts were further screened for the mentioned data and after that, we performed data extraction in an Excel sheet. Any disagreements during data extraction and study selection were discussed with a third reviewer, who was an expert in this field.

 We extracted the data for the following variables: first author’s name, year of publication, definitive diagnosis of patients with renal dysfunction, the gold standard for the definitive diagnosis of RTD, number of the control group, number of patients with kidney transplant dysfunction, mean and standard deviation (SD) of RI in the control group and mean and SD of RI in patients with renal allograft dysfunction. 

**Study appraisal:** The quality of studies was evaluated based on the New Castle Ottawa Scale (NOS) by two reviewers, and any disagreements during data extraction and study selection were discussed with a fourth reviewer (15).

**Data synthesis:** We used a *meta* package in the R statistical software (V.4.0.5) for the analysis of our data (16). The pooled mean of RI was calculated for both the control group and patients with renal allograft dysfunction. To examine the heterogeneity of the included studies, we used the I^2^ statistic. If heterogeneity was greater than 75%, it would be considered significant heterogeneity, and the random-effects model was used. (17) The pooled mean for RI was calculated with a 95% confidence interval. To display the result of our meta-analysis, we used the forest plot with the pooled effect and mean for each study. Finally, we used a t-test to compare the two pooled means (SD). We utilized the Egger test to assess the potential publication bias in our study along with the funnel plot visualized by STATA Version 17.

## Results

**Figure 1:**
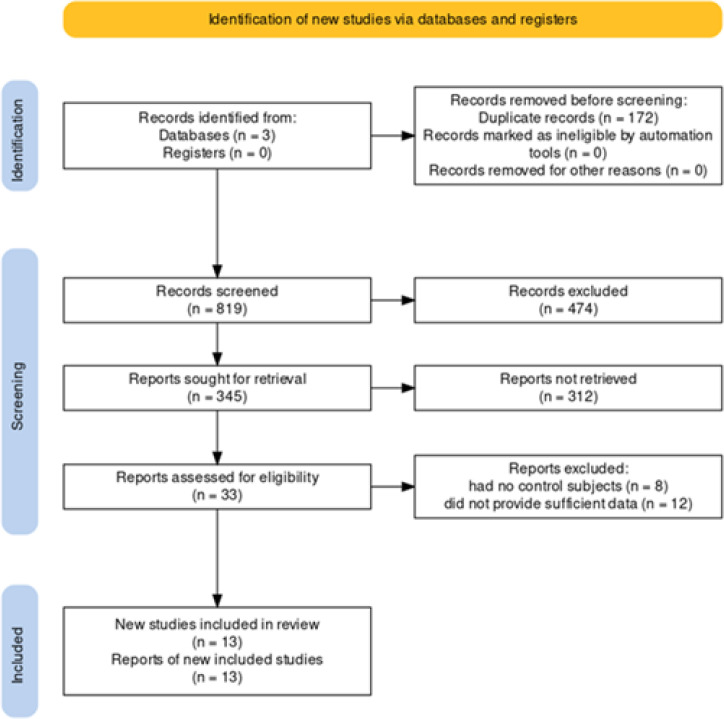
Summary of the study selection process

We summarized the study selection process in figure 1. After removing the duplicated studies, 819 records remained. The second phase was title/abstract screening, after which 35 studies remained to be evaluated with their full text. We excluded 22 studies due to the following exclusion criteria 1) 12 studies did not provide sufficient data, 2) 8 studies had no control subjects. Finally, 13 studies were totally eligible for our study. Table 1 summarizes the main findings from each study.

**Table 1:** Summary of the main findings of the included studies

**Author**	**Year**	**Diagnosis**	**Gold standard of diagnosis**	**Number of patients**	**Number of control group**	**Mean of RI in patients (SD)**	**Mean of RI in control d (SD)**
**Chow (18)**	2001	Acute rejection	Biopsy	47	49	0.8 (0.11)	0.82 (0.1)
**Morais (19)**	2003	Transplant renal artery stenosis	MR angiography and digital subtraction arteriography	10	19	0.62 (0.1)	0.67 (0.07)
**Dupont (20)**	2003	Acute rejection	Biopsy	68	91	0.73 (0.11)	0.74 (0.11)
**Drudi (21)**	2004	Acute rejection	Biopsy	110	213	0.85 (0.14)	0.68 (0.02)
		Chronic rejection	Biopsy	171	213	0.75 (0.09)	0.68 (0.02)
**Sharma (22)**	2004	Acute rejection	Biopsy	462	278	0.83 (0.15)	0.69 (0.09)
**Datta (23)**	2005	Acute rejection	Biopsy	24	6	0.93 (0.29)	0.98 (0.39)
**Župunski (24)**	2005	Renal Transplant Artery Stenosis	Biopsy	34	34	0.62 (0.1)	0.68 (0.07)
**Gao (25)**	2011	Interstitial fibrosis, tubular atrophy, and vascular/glomerular sclerosis	Biopsy	79	34	0.77 (0.05)	0.78 (0.05)
**Rigler (26)**	2013	ATN and/or Acute rejection	Biopsy	40	28	0.83 (0.03)	0.82 (0.04)
**Gao (27)**	2015	Interstitial Fibrosis/Tubular Atrophy	Biopsy	10	12	0.75 (1.78)	0.69 (0.08)
**Abd El-Motaal (28)**	2019	Acute tubular injury	Biopsy	13	13	0.76 (0.03)	0.63 (0.06)
		Chronic allograft injury	Biopsy	14	13	0.66 (0.05)	0.63 (0.06)
**Qi (29)**	2020	Renal Transplant Artery Stenosis	Biopsy	16	16	0.51 (0.1)	0.67 (0.13)
**Goyal (30)**	2021	Acute rejection	Biopsy	5	86	0.6 (0.1)	0.6 (0.1)
		Acute tubular necrosis	Biopsy	12	86	0.7 (0.1)	0.6 (0.1)

We included 13 studies consisting of 1191 control cases and 1115 patients with kidney transplant dysfunction. As some studies had divided subpopulations of RTD, 16 different cohorts of patients were included in our data synthesis. 

**Figure 2:**
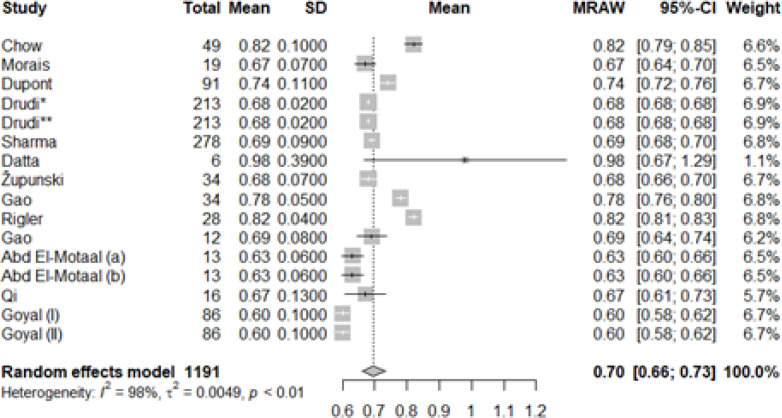
Mean of resistive index (RI) in individual studies and pooled mean in the control group (forest plot)

**Figure 3:**
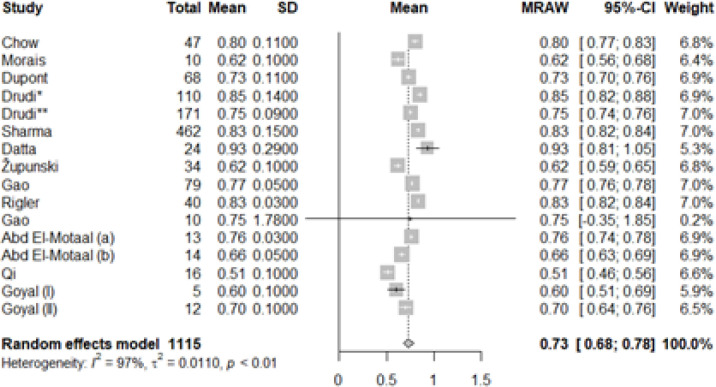
Mean of resistive index (RI) in individual studies and pooled mean in patients with RTD. (Drudi*: acute rejection, Drudi**: chronic rejection, a: acute tubular injury, b: Chronic allograft injury, I: Acute rejection, II: Acute tubular necrosis)

The mean of RI in the control group ranged from 0.60 to 0.98 and the mean of RI in patients with kidney transplant dysfunction ranged from 0.51 to 0.93. The pooled mean (95% CI) for the control group was calculated to be 0.71 (0,67, 0.75) (figure 2) and for patients with renal transplant dysfunction was 0.73 (0.68, 0.78) (figure 3). 

The most studied pathologies were acute rejection followed by renal artery stenosis. The gold standard for diagnosis of renal transplant dysfunction in all studies except one was a biopsy.

**Figure 4:**
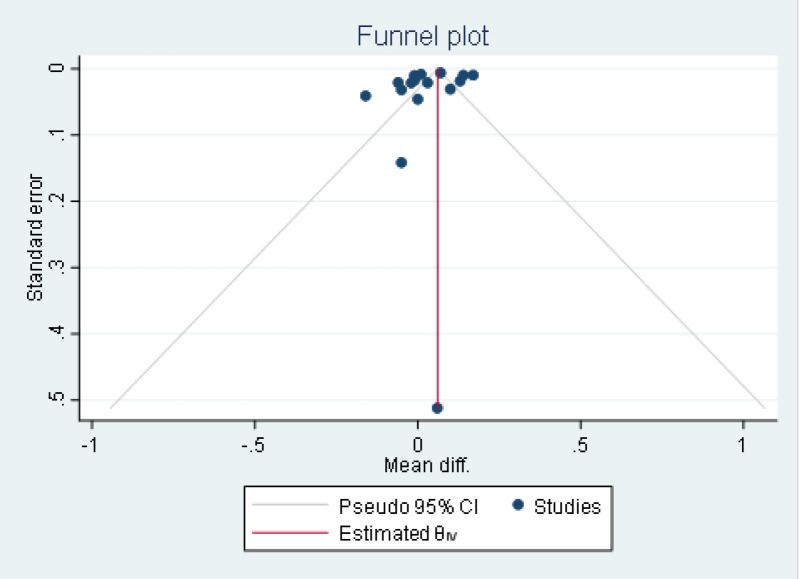
A funnel plot of all of the studies' mean for RI

The pooled mean effect showed a statistically significant difference between the control group and RTD patients (p< 0.05). As shown in figure 4, there was no potential publication bias in the study due to the symmetry of the plot and Egger test was not significant (P=0.329), showing no publication bias.

**Quality assessment:** Table 2 shows the quality of the studies included in the analysis based on the New Castle Ottawa Scale. The table includes 12 studies and each row represents a study. 

The columns are divided into three categories: Selection, Ascertainment of Exposure, and Non-Response Rate. The table suggests that the included studies have a low risk of bias and proper method according to the New Castle Ottawa Scale. 

**Table 2:** Quality of studies based on New Castle Ottawa Scale. * represent low risk of bias and proper method

	**SELECTION**				**Ascertainment of Exposure**	**Non-Response Rate**
**Author**	**Is the Case Definition Adequate?**	**Representativeness of the Cases**	**Selection of Controls**	**Definition of Controls**		
**Chow (18)**	*	*	*	*	*	*
**Morais (19)**	*	*	*	*	*	-
**Dupont (20)**	*	*	*	*	*	*
**Drudi (21)**	*	*	*	*	*	*
**Sharma (22)**	*	*	*	*	*	*
**Datta (23)**	*	*	*	*	*	*
**Župunski (24)**	*	*	*	*	*	*
**Gao (25)**	*	-	*	*	*	-
**Rigler (26)**	*	*	*	*	*	*
**Gao (27)**	*	*	*	*	*	*
**Abd El-Motaal (28)**	*	*	*	*	*	*
**Qi (29)**	*	*	*	*	*	*
**Goyal (30)**	*	-	*	*	*	-

## Discussion

Our systematic review and meta-analysis study demonstrated a significant difference in the resistive index (RI) between patients with renal transplant dysfunction and the control group. Our findings support the use of Doppler sonography parameters, including RI, as an essential tool for assessing RTD. Greyscale ultrasonography (US), color Doppler ultrasonography (DUS), and contrast-enhanced ultrasonography (CEUS) are examples of US assessment techniques that may be used to assess the morphology and vascular health of an allograft. With the hope of utilizing a noninvasive technology that can detect early renal hemodynamic abnormalities and resultant transplant failure, several experiments have been performed on DUS parameters, with the majority focusing on the RI (18-30). Some studies have reported positive results utilizing the RI as a beneficial tool to monitor renal disorders, whereas others have reported negative results when using the RI to assess renal illnesses. Our study showed that there is a significant difference between the RI in patients with renal transplant dysfunction and the control group. According to one study, the location of renal histopathologic alterations mattered more than the severity of changes in causing a higher RI (31). This result was in line with another study, which indicated that disorders of the tubulointerstitial compartment resulted in a higher RI, but diseases affecting only the glomeruli did not (7) .

The RI is measured by subtracting the peak systolic velocity (PSV) and end-diastolic velocity (EDV) divided by the PSV. In theory, a nonproportional change in the PSV, EDV, or both might modify the RI. In clinical practice, various appearances of the DUS waveform and velocity in the interlobar arteries can be caused by changes in renal hemodynamic factors due to alterations in extra or intrarenal conditions. Various extrarenal factors can influence renal blood flow and significantly alter the DUS waveform. These factors include the function of the right side of the heart, cardiac output, blood pressure, and the vascular status of the recipient (32).

In the study conducted by Cano et al., it has been reported that RI is a valuable marker in assessing kidney transplant function. More than 0.7 increase can alert for acute kidney allograft dysfunction. However, it is unable to distinguish between other possible conditions such as acute or chronic rejection, tubular necrosis, renal thrombosis, ureteral obstruction, infection, or medication toxicity (33). Several studies have used RI and PI to effectively evaluate the long-term outcomes instantly following the transplantation (34, 35). One study used RI in various transplanted organs, such as kidney grafts, and showed that RI is mostly valuable for detecting systemic vascular pathologies and prediction of death after transplant rather than the diagnosis of specific renal graft dysfunctions (36). Similarly, Heine et al. discovered that a higher RI was linked to subclinical systemic atherosclerosis, high pulse pressure, and diabetes. They could not establish a link between RI and GFR of the kidney (11). Other indices such as PSV and EDV in the interlobar artery should be taken into account when evaluating DUS values and examining the relationships among these measures, clinical presentation, or laboratory test results using DUS to examine kidney transplant hemodynamics. The limitations of our study are as follows: 1) we did not include all doppler ultrasonography parameters; 2) In our study, we did not stratify the data based on histopathological changes. However, the latter is directly caused by a low number of studies in each category. To the best of our knowledge, this is the first meta-analysis comparing the sonographic parameters in patients with kidney allograft dysfunction and control groups. 

Our study showed that there is a significant difference between RI in patients with kidney transplant dysfunction and the control group. However, our research suggests that RI cannot substitute kidney biopsy in the management and diagnosis of renal transplants dysfunction. More research on Doppler parameters with the potential to be used in noninvasive kidney transplant hemodynamics should be encouraged.

## References

[1] Perrella RR, Duerinckx AJ, Tessler FN (1990). Evaluation of renal transplant dysfunction by duplex Doppler sonography: a prospective study and review of the literature. Am J Kidney Dis.

[2] Meier M, Fricke L, Eikenbusch K (‎‎2017). The serial duplex index ‎improves differential diagnosis of acute renal transplant dysfunction. J Ultrasound Med.

[3] Hollis E, Shehata M, Khalifa F (2017). Towards non-invasive ‎diagnostic techniques for early detection of acute renal transplant rejection: A review. Egypt J Radiol Nucl Med.

[4] Gao J, Ng A, Shih G (2007). Intrarenal color duplex ultrasonography: a window to vascular complications of renal transplants. J Ultrasound Med.

[5] Leong KG, Coombs P, Kanellis J (2015). Renal transplant ultrasound: The nephrologist's perspective. Australas J Ultrasound Med.

[6] Park SB, Kim JK, Cho KS (2007). Complications of renal transplantation: ultrasonographic evaluation. J Ultrasound Med.

[7] Platt JF, Rubin JM, Ellis JH (1997). Lupus nephritis: predictive value of conventional and Doppler US and comparison with serologic and biopsy parameters. Radiology.

[8] Platt JF (1997). Doppler ultrasound of the kidney. Semin Ultrasound CT MR.

[9] Khosroshahi HT, Tarzamni M, Oskuii RA (2005). Doppler ultrasonography before and 6 to 12 months after kidney transplantation. Transplant Proc.

[10] Rivolta R, Castagnone D, Elli A, Di Palo FQ (1996). Evaluation of kidney graft function by arterial flow using colour Doppler flowmetry. Euro J Ultra.

[11] Heine GH, Gerhart MK, Ulrich C, Kaler H, Girndt M (2005). Renal Doppler resistance indices are associated with systemic atherosclerosis in kidney transplant recipients. Kidney Int.

[12] Krumme B, Grotz W, Kirste G, Schollmeyer P, Rump LC (1997). Determinants of intrarenal Doppler indices in stable renal allografts. J Am Soc Nephrol.

[13] Krumme B (2006). Renal Doppler sonography–update in clinical nephrology. Nephron Clin Pract.

[14] Moher D, Liberati A, Tetzlaff J, Altman DG, Group P (2009). Preferred reporting items for systematic reviews and meta-analyses: the PRISMA statement. PLoS Med.

[15] Luchini C, Stubbs B, Solmi M, Veronese N (2017). Assessing the quality of studies in meta-analyses: Advantages and limitations of the Newcastle Ottawa Scale. World J Metaanal.

[16] Schwarzer G, Carpenter JR, Rücker G (2015). Meta-analysis with R.

[17] DerSimonian R, Kacker R (2007). Random-effects model for meta-analysis of clinical trials: an update. Contemp Clin Trials.

[18] Chow L, Sommer FG, Huang J, Li KC (2001). Power Doppler imaging and resistance index measurement in the evaluation of acute renal transplant rejection. J Clin Ultrasound.

[19] De Morais RH, Muglia VF, Mamere AE (2003). Duplex Doppler sonography of transplant renal artery stenosis. J Clin Ultrasound.

[20] Dupont PJ, Dooldeniya M, Cook T, Warrens AN (2003). Role of duplex Doppler sonography in diagnosis of acute allograft dysfunction-time to stop measuring the resistive index?. Transpl Int.

[22] Sharma AK, Rustom R, Evans A (2004). Utility of serial Doppler ultrasound scans for the diagnosis of acute rejection in renal allografts. Transpl Int.

[23] Datta R, Sandhu M, Saxena AK (2005). Role of duplex Doppler and power Doppler sonography in transplanted kidneys with acute renal parenchymal dysfunction. Australas Radiol.

[24] Zupunski A, Buturović-Ponikvar J (2005). Duplex-Doppler long-term follow-up of renal transplant artery stenosis: case controlled study. Ther Apher Dial.

[25] Gao J, Rubin JM, Xiang DY (2011). Doppler Parameters in renal transplant dysfunction correlations with histopathologic changes. J Ultrasound Med.

[26] Rigler AA, Vizjak A, Ferluga D, Kandus A, Buturovic-Ponikvar J (2013). Ultrasonography parameters and histopathology findings in transplanted kidney. Transplant Proc.

[27] Gao J, Rubin JM, Weitzel W (2015). Comparison of ultrasound corticomedullary strain with Doppler parameters in assessment of renal allograft interstitial fibrosis/tubular atrophy. Ultrasound Med Biol.

[28] El-Motaal AM, Dawoud RM, Sherif MF, Eldiasty TA (2019). Role of ultrasound, Color duplex Doppler and sono-elastography in the evaluation of renal allograft complications. Egypt J Radiol Nucl Med.

[29] Qi RC, Qi GS, Zhu D, Wang JN (2020). Diagnosis and treatment of early transplant renal artery stenosis: Experience from a center in Eastern China. Transplant Proc.

[30] Goyal A, Hemachandran N, Kumar A (2021). Evaluation of the Graft Kidney in the Early Postoperative Period: Performance of Contrast-Enhanced Ultrasound and Additional Ultrasound Parameters. J Ultrasound Med.

[31] Galešić K, Sabljar-Matovinović M, Tomić M, Brkljačić B (2004). Renal vascular resistance in glomerular diseases–correlation of resistance index with biopsy findings. Coll Antropol.

[32] Parolini C, Noce A, Staffolani E (2009). Renal resistive index and long-term outcome in chronic nephropathies. Radiology.

[33] Cano H, Castañeda D, Patiño N (2014). Resistance index measured by Doppler ultrasound as a predictor of graft function after kidney transplantation. Transplant Proc.

[34] Buturovic-Ponikvar J, Cerne S, Arnol M (2010). Early kidney graft size and Doppler parameters are associated with kidney graft function 1 year after transplantation. Transplant Proc.

[35] Barba J, Rioja J, Robles JE (2011). Immediate renal Doppler ultrasonography findings (< 24 h) and its association with graft survival. World J Urol.

[36] Seiler S, Colbus SM, Lucisano G (2012). Ultrasound renal resistive index is not an organ-specific predictor of allograft outcome. Nephrol Dial Transplant.

